# Athlete-focused eating disorder programming in higher levels of care: feasibility and clinical outcomes from a naturalistic setting

**DOI:** 10.3389/fnut.2026.1734726

**Published:** 2026-05-12

**Authors:** Eva-Molly Petitto Dunbar, Madeline Palermo, Sophie R. Abber, Leslie K. Anderson, Christina R. Felonis, Anna M. Karam Jones, Brittany K. Bohrer, Christina E. Wierenga, Roxanne E. Rockwell, Kimberly Claudat

**Affiliations:** 1 Department of Psychiatry, Eating Disorder Treatment and Research Program, University of California San Diego, San Diego, CA, United States; 2 Department of Pediatrics, Rady Children’s Hospital, University of California San Diego School of Medicine, San Diego, CA, United States

**Keywords:** athletes, dysfunctional exercise, eating disorders, higher level of care, treatment outcomes

## Abstract

Eating disorders (EDs) are a significant public health concern associated with psychiatric, physical, and functional impairments. Athletes experience elevated ED prevalence compared to non-athletes, with rates up to 19% in men and 45% in women. Among athletes, EDs present additional concerns including increased injury risk, impaired performance, prolonged illness, and potential premature athletic career termination. Dysfunctional exercise—a maladaptive pattern characterized by rigid rules, guilt, compulsivity, and inability to stop despite harm—is common in this population and linked to poorer outcomes. Despite increasing recognition of these risks, few studies have systematically evaluated athlete-focused interventions in higher levels of care. This study examined the feasibility and preliminary effectiveness of specialized athlete programming within partial hospitalization and intensive outpatient ED treatment programs. Participants included 182 adolescent and adult patients who self-identified as athletes and attended at least four athlete-focused therapy groups. Outcomes included dysfunctional exercise symptoms (EPSI), ED symptoms (EDE-Q), ED-related impairment (CIA), depression (PHQ-9), and anxiety (STAI). Linear mixed-effects models evaluated changes across admission, mid-treatment, discharge, and 6- and 12-month follow-ups. Pairwise comparisons of estimated marginal means were conducted using Tukey-adjusted contrasts to identify significant decreases between specific time points. Significant decreases were observed in dysfunctional exercise, ED symptoms, ED-related impairment, depression, and anxiety. The steepest improvements occurred between admission and mid-treatment for all outcomes except anxiety, which remained stable at first and declined sharply by discharge; all outcomes showed stabilization through 12-month follow-up. Findings provide preliminary empirical support for the feasibility and clinical utility of integrating athlete-focused programming into higher-level ED care, highlighting the importance of addressing the unique challenges and treatment needs of athletes.

## Introduction

Eating disorders (EDs) represent a significant public health concern, associated with significant emotional, physical, social, and financial burden, as well as high rates of psychiatric comorbidity (1). Dysfunctional exercise (i.e., a pathological relationship with exercise that results in negative physical and/or psychological health consequences) is prevalent among individuals with EDs, with approximately 48% engaging in dysfunctional exercise at the start of treatment, and with lifetime prevalence estimates ranging from 63% to 86% (2, 3). Dysfunctional exercise is an overarching construct that captures maladaptive patterns of exercise, including compulsive exercise, exercise dependence, and exercise addiction (4–7). Dysfunctional exercise is best understood not simply in terms of exercise frequency or duration, but also by the maladaptive cognitions that drive it. These include rigid and inflexible rules, compulsivity, affect-regulation challenges, punitive or compensatory attitudes, and feelings of guilt when exercise is missed, alongside a perceived or demonstrated inability to stop despite awareness of potential harm (3, 5, 8). This cognitive pattern is critically important to address, as it has been strongly associated with both the onset and maintenance of EDs, with greater disturbance at baseline associated with poorer treatment outcomes (9), and greater likelihood of relapse (10). Research indicates that evidence-based prevention and treatment approaches can help lessen both the severity and overall burden of EDs (11), but consensus on how to approach dysfunctional exercise in ED treatment has not been established, particularly in higher-level-of-care settings.

This challenge is amplified within athletic populations, where a culture of intense physical activity and pressure for specific body compositions may increase vulnerability to dysfunctional exercise. Athletes with EDs often share motivations for exercise engagement seen in non-athletes, such as weight and shape concerns or emotion regulation, and may also face unique pressures to continue training or competing despite their illness (12). The syndrome Relative Energy Deficiency in Sport (RED-S) further characterizes health risks and potential consequences of energy deficiency resulting from EDs, highlighting risks such as impaired physiological functioning, reduced performance, and long-term health consequences (13, 14). Although prevalence rates of EDs and disordered eating among athletes vary, reports suggest approximately 30%–45% of female athletes and 8%–15% of male athletes experience disordered eating (15, 16). Among elite athlete cohorts, these rates may reach 45% in female athletes and 19% in male athletes (15). Athletes engaged in lean sports—where a low body weight or lean physique is emphasized for performance or aesthetics, such as gymnastics, figure skating, distance running, and dance —face particularly heightened risk, with some estimates of disordered eating prevalence as high as 61% (17–19). Importantly, for the athlete, an ED not only presents adverse health consequences but also significantly increases injury risk and impairs performance (20, 21).

Athletes represent a high-risk population, and present to ED treatment with unique complexities related to their athlete identity, performance pressures, and the role of exercise in their life. Standard ED care often does not provide specialized programming for athletes and prescribes exercise abstinence throughout the course of treatment, which can lead to significant treatment resistance, under-reporting of dysfunctional exercise behaviors, and early dropout for athletes. This approach fails to address the psychological complexities of their mindset or the importance of planning for a medically safe return-to-sport that supports psychological and physical readiness, including injury prevention (22). For example, qualities traditionally valued in sport, such as mental toughness, drive for excellence, and performing despite pain may be challenging to disentangle from qualities that can fuel ED symptomatology, complicating recovery if not addressed (12). A substantial gap remains in the availability of optimal treatment interventions, and there is a distinct lack of systematic measurement of the effectiveness of existing treatments specifically tailored for this population. This highlights a critical need to develop and evaluate evidence-based approaches to safely and effectively address dysfunctional exercise as a key component of ED recovery for athletes.

Extant literature and clinical guidelines recommend a stage-based, individualized approach to exercise reintegration, contingent on medical, psychological, and nutritional stability. This model, supported by multidisciplinary teams with dual expertise in EDs and sports medicine, aims to transition athletes from dysfunctional exercise to functional, joyful physical activity through progressive stages of monitored exercise incorporation. Importantly, studies have shown that psychological recovery may lag behind physical stability (23, 24), underscoring the need for targeted psychological strategies for athletes. Proposed approaches highlight components such as psychoeducation about dysfunctional versus functional exercise, exploration of athlete identity, and sports nutrition as important for supporting a safe return-to-sport (22). Despite the growing clinical practice of athlete-specific interventions, there remains a dearth of empirical data evaluating the efficacy of these specialized interventions in reducing dysfunctional exercise behaviors and improving ED treatment outcomes. This study aims to address this gap by examining the treatment outcomes of a specialized athlete-programming model.

Thus, the present study aimed to: (1) characterize demographic characteristics of patients participating in athlete programming during intensive treatment, and (2) examine changes in exercise symptoms, ED symptoms, ED-related impairment, and comorbid symptoms of depression and anxiety throughout treatment and at follow-up. We hypothesized that participation in athlete programming during treatment would be associated with reductions in dysfunctional exercise symptoms, ED symptoms, ED-related impairment, and depression and anxiety symptoms. Further, we expected that these gains would be maintained during the follow-up period.

## Methods

### Participants and procedure

The present analyses included adolescents and adults seeking ED treatment at the partial hospitalization level of care between May 2019 and January 2025. Program admission criteria aligned with American Psychiatric Association guidelines (25). Data were obtained from a larger study examining outcomes from this treatment program (26, 27). Patients were offered the opportunity to participate in the study at admission. Following informed consent or parental consent plus assent, participants completed measures within 14 days of admission (i.e., Admission), 1 month into treatment (i.e., Mid-treatment), within 14 days of discharge (i.e., Discharge), 6 months following discharge (i.e., 6MFU) and 12 months following discharge (i.e., 12MFU). Data from participants who attended at least four Athlete therapy groups were included in the present analyses, as this threshold was selected to ensure meaningful exposure to core curriculum content, resulting in 182 adolescents and adults with complete data at admission. Approximately 91.4% of the sample completed the Mid-treatment assessment (*n* = 170), 83.3% completed the Discharge assessment (*n* = 155), 55.4% completed the 6MFU assessment (*n* = 103), and 43% completed the 12MFU assessment (*n* = 80). All procedures were approved by the University Institutional Review Board (#180055).

### Athlete group

Patients were eligible to participate in the Athlete group if they: (a) self-identified as an athlete or endorsed athletic identity on the Athletic Identity Measurement Scale (AIMS; 28); if athletic identity was indicated on the AIMS, the treatment team further explored athletic identity with the patient and whether participation in the group would be appropriate; (b) were planning for, exploring, or considering a return to sport or a high level of physical activity (e.g., collegiate, semi-professional, or professional athletics, marathon training, coaching) following discharge from the treatment program; and (c) had approval from their full treatment team (i.e., medical providers, dietitian, and therapist). Eligible patients could opt into specialized athlete programming, including a weekly Athlete group.

The Athlete group curriculum included psychoeducation and discussion about exercise, RED-S, athlete identity, body-image pressures in sport, attitudes toward exercise, sports nutrition, and communication with coaches and teammates. Additional components emphasized fostering body awareness, building the ability to rest, developing coping strategies beyond exercise, challenging expectations about performance during return to sport, and addressing relapse prevention in the athletic environment.

Additional exercise-related programming (e.g., a group providing psychoeducation and exposure and response prevention related to dysfunctional exercise) was available to all patients regardless of athlete status (see 26, 27, respectively for details on general adolescent and adult programming). However, our analyses focused specifically on the Athlete group, and participation in other programming was not tracked in this sample.

### Measures

#### Diagnostic interviews

Semi-structured interviews administered by trained bachelor’s-level research assistants and doctoral-level trainees were used for ED diagnosis. Adult patients were administered the Structured Clinical Interview for *DSM-5* (SCID-5; 29), and adolescent patients were administered the Schedule for Affective Disorders and Schizophrenia for School Age Children (KSADS; 30). Interviewers received extensive training in clinical interviews. Supervision was provided by licensed clinical psychologists with expertise in assessment of EDs and diagnoses were confirmed via regular consensus meetings.

#### Dysfunctional exercise symptoms

The Eating Pathology Symptoms Inventory (EPSI) Excessive Exercise subscale measured dysfunctional exercise symptoms (31). The five items were rated on a scale of zero (“Never”) to four (“Very Often”) and summed into a subscale score with higher scores indicating greater dysfunctional exercise symptoms (range = 0–20): “I felt that I needed to exercise nearly every day;” “I pushed myself extremely hard when I exercised;” “I planned my days around exercising;” “I engaged in strenuous exercise at least 5 days per week;” and “I exercised to the point of exhaustion.” Internal consistency of the subscale was good across time points (α = 0.89–0.93).

#### ED symptoms

The Eating Disorders Examination Questionnaire (EDE-Q) Global scale measured ED symptoms (32). The 28 items were rated on a scale of zero to six and averaged into a Global score (range = 0–6), with higher scores reflecting greater ED symptoms. Internal consistency of the Global scale was good across time points (α = 0.82–0.92).

#### ED-related impairment

The 16-item Clinical Impairment Assessment (CIA) measured ED-related impairment (33). Items were rated on a scale of zero (“not at all”) to three (“a lot”) with higher scores reflecting greater impairment (range = 0–48). Internal consistency was good across time points (α = 0.94–0.96).

#### Depression symptoms

The Patient Health Questionnaire-9-item (PHQ-9) measured depression symptoms (34). Items were rated on a scale of zero (“not at all”) to three (“nearly every day”) with higher scores reflecting greater depression symptoms (range = 0–27). Internal consistency was good across time points (α = 0.87–0.91).

#### Anxiety symptoms

The 20-item State-Trait Anxiety Inventory (STAI) Trait subscale measured anxiety symptoms (35). Items were rated on a scale of one (“not at all”) to four (“very much so”) with higher scores reflecting greater anxiety symptoms (range = 20–80). Positive statements are reverse-scored. Internal consistency was good across time points (α = 0.89–0.92).

### Statistical analysis

Analyses were conducted in R using lme4 (36) and emmeans (37) packages with significance evaluated at *p* < 0.05. Data were examined for normality. Descriptive statistics were used to characterize the demographics of the sample. Linear mixed effects models examined change over time in dysfunctional exercise symptoms, ED symptoms, ED-related impairment, depression, and anxiety. In all models, time was treated as a categorical predictor to allow for non-linear trajectories. Models included random intercepts for participants to account for within-subject correlations across five measurement occasions (i.e., Admission, Mid-treatment, Discharge, 6MFU, and 12MFU). This flexible approach estimates separate means at each time point and does not assume linear change over time. Pairwise comparisons of estimated marginal means were conducted using Tukey-adjusted contrasts to identify significant decreases between specific time points. Standardized effect sizes (Cohen’s *d*; 38) were calculated for pairwise comparisons and interpreted as small (0.2), medium (0.5), or large (0.8).

#### Missing data analyses

Initial sensitivity analyses compared participants with complete versus missing data at each time point. At Mid-treatment, 6MFU, and 12MFU, missingness varied by certain demographic predictors, although effect sizes were small. At Discharge, participants with missing data differed significantly from those without missing data, showing moderately higher baseline EDE-Q Global, EPSI-Excessive Exercise, and CIA scores, as well as differences in admit BMI and ED diagnosis (Supplementary Table 1). Given that these findings suggest data at discharge may be not missing at random, we conducted pattern mixture models to examine whether participants missing data at discharge differed in baseline values or longitudinal trajectories compared to those with complete data. Results (see Supplementary material) indicated that for the EDE-Q, participants with missing data at discharge showed larger decreases in symptoms over time. This suggests that the missingness did not bias results toward inflated symptom levels. For the EPSI-Excessive Exercise and CIA models, trajectories were similar across groups, indicating that the missing data were unlikely to bias the findings. Additionally, comparisons between participants who completed versus did not complete the 6MFU and 12MFU surveys indicated no significant differences on baseline demographic or clinical variables with the exception of admission BMI at 12MFU. Specifically, individuals who completed the 12MFU had a slightly lower admission BMI than those who did not complete the 12MFU. Taken together, these analyses support the appropriateness of using linear mixed effects models with Full Information Maximum Likelihood (FIML) to handle missing data in the primary analyses.

## Results

### Characteristics of patients participating in athlete programming

Adolescent and adult participants with data at admission (*N* = 182) were included in analyses. On average, these participants were young adults, primarily female-identifying and Non-Hispanic White, with anorexia nervosa—restricting type (Table 1).

**TABLE 1:** Demographic information for study participants across all five time points.

	Admission (*N* = 182)	Mid-treatment (*N* = 171)	Discharge (*N* = 155)	6MFU (*N* = 103)	12MFU (*N* = 80)
Variable					
Age	19.05 (6.83)	18.64 (6.49)	19.13 (7.04)	18.19 (4.73)	18.19 (4.54)
Gender					
Boy/man	22 (12.1%)	18 (10.5%)	16 (10.3%)	7 (6.8%)	5 (6.3%)
Girl/woman	155 (85.2%)	149 (87.1%)	137 (88.4%)	94 (91.3%)	74 (92.5%)
Trans-man/trans-masculine	1 (0.5%)	1 (0.6%)	0 (0%)	0 (0%)	0 (0%)
Trans-woman/trans-feminine	0 (0%)	0 (0%)	0 (0%)	0 (0%)	0 (0%)
Genderqueer/gender non-conforming/non-binary	2 (1.1%)	2 (1.2%)	2 (1.3%)	1 (1.0%)	0 (0%)
BMI	19.42 (3.00)	20.11 (3.03)	21.15 (2.98)	20.94 (2.63)	21.11 (3.05)
Race					
White	153 (84.1%)	146 (85.4%)	133 (85.8%)	90 (87.4%)	68 (85.0%)
Asian	11 (6.0%)	8 (4.7%)	8 (5.2%)	5 (4.9%)	5 (6.3%)
Black	0 (0%)	0 (0%)	0 (0%)	0 (0%)	0 (0%)
Native Hawaiian or Pacific Islander	0 (0%)	0 (0%)	0 (0%)	0 (0%)	0 (0%)
Native American or Alaskan native	0 (0%)	0 (0%)	0 (0%)	0 (0%)	0 (0%)
Other	16 (8.8%)	15 (8.8%)	12 (8.4%)	7 (6.8%)	7 (8.8%)
Ethnicity					
Hispanic	26 (14.3%)	25 (14.6%)	18 (11.6%)	16 (15.5%)	12 (15.0%)
Non-Hispanic	155 (85.2%)	145 (84.8%)	136 (87.7%)	87 (84.5%)	67 (83.8%)
EDE-Q Global	3.57 (1.50)	3.00 (1.45)	2.24 (1.47)	2.31 (1.55)	2.12 (1.51)
EPSI-Excessive Exercise	11.18 (6.91)	8.01 (6.56)	5.19 (5.30)	6.63 (5.91)	6.45 (6.26)
Clinical Impairment Assessment	30.46 (11.25)	28.00 (11.62)	19.42 (12.35)	17.87 (12.58)	15.96 (12.49)
PHQ-9	12.88 (10.45)	12.08 (6.73)	8.71 (6.16)	9.19 (6.63)	8.65 (6.70)
STAI-Trait	59.87 (10.45)	59.33 (10.34)	53.63 (10.74)	52.02 (12.52)	50.47 (13.09)
ED diagnosis					
AN-R	98 (53.8%)	96 (56.1%)	92 (59.4%)	132 (58.4%)	53 (66.3%)
AN-BP	28 (15.4%)	26 (15.2%)	25 (16.1%)	64 (62.1%)	10 (12.5%)
BN	15 (8.2%)	12 (7.0%)	11 (7.1%)	5 (4.9%)	3 (3.8%)
ARFID	10 (5.5%)	9 (5.3%)	9 (5.8%)	5 (4.9%)	5 (6.3%)
OSFED	31 (17.0%)	28 (16.4%)	18 (11.6%)	12 (11.7%)	9 (11.3%)

Gender, race/ethnicity, and ED diagnosis were only collected at Admission. BMI was based on measured height and weight at Admission, Mid-treatment, and Discharge, and was based on EDE-Q responses at 6MFU and 12MFU. AN-R, anorexia nervosa, restricting type; AN-BP, anorexia nervosa, binge/purge type; BN, bulimia nervosa; ARFID, avoidant/restrictive food intake disorder; OSFED, other specified feeding or eating disorder.

### Outcomes for patients in athlete programming

#### Dysfunctional exercise symptoms

**FIGURE 1:**
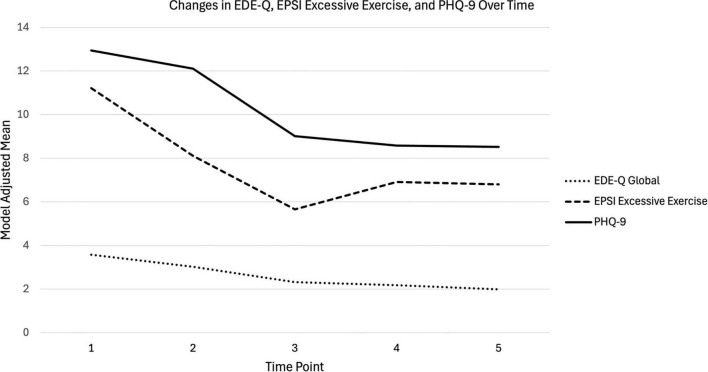
Model-adjusted means for Eating Disorders Examination Questionnaire (EDE-Q) Global, Eating Pathology Symptoms Inventory (EPSI) Excessive Exercise, and Patient Health Questionnaire-9-item (PHQ-9) scores across five time points (Admission, Mid-Treatment, Discharge, 6-Month Follow-Up, and 12-Month Follow-Up). Values represent estimated marginal means from linear mixed-effects models with random intercepts.

Linear mixed-effects modeling revealed significant changes in dysfunctional exercise symptoms over time. Estimated marginal means indicated a decrease from Admission (M = 11.22, 95% CI [10.29, 12.14]) to Discharge (M = 5.66, 95% CI [4.67, 6.64], Figure 1), followed by a non-significant increase and stabilization at later time points (6MFU: M = 6.91, 95% CI [5.77, 8.05]; 12MFU: M = 6.80, 95% CI [5.54, 8.06], Figure 1). Pairwise comparisons revealed significant decreases from Admission to all time points (all *p*s < 0.0001, *d*_admission–mid–treatment_ = 0.68, *d*_admission–discharge_ = 1.22, *d*_admission–6MFU_ = 0.95, d_admission–12MFU_ = 0.97) and from Mid-treatment to Discharge (*p* < 0.0001, *d* = 0.54). No significant differences were observed among Mid-treatment, 6MFU, and 12MFU, or between Discharge, 6MFU, and 12MFU after adjustment for multiple comparisons. These results suggest a sharp initial decrease in dysfunctional exercise symptoms through Discharge, followed by a leveling off in subsequent assessments.

#### ED symptoms

Linear mixed-effects modeling revealed significant changes in ED symptoms over time. Estimated marginal means decreased steadily from Admission (M = 3.58, 95% CI [3.37, 3.80]) to 12MFU (M = 1.99, 95% CI [1.72, 2.27], Figure 1). Pairwise comparisons indicated significant reductions from Admission to all subsequent time points (all *ps* < 0.0001, *d*_admission–mid–treatment_ = 0.61, *d*_admission–discharge_ = 1.37, *d*_admission–6MFU_ = 1.51, *d*_admission–12MFU_ = 1.72). Additionally, there were significant reductions from Mid-Treatment to all other time points (all *ps* < 0.0001, *d*_mid–treatment_*_–_*_discharge_ = 0.70, *d*_mid–treatment–6MFU_ = 0.84, *d*_mid–treatment––12MFU_ = 1.03). Differences between later time points (Discharge, 6MFU, and 12MFU) were not statistically significant, suggesting a plateau in scores after Discharge. These results suggest a non-linear trajectory of ED symptoms characterized by rapid initial improvement followed by stabilization.

#### ED-related impairment

**FIGURE 2:**
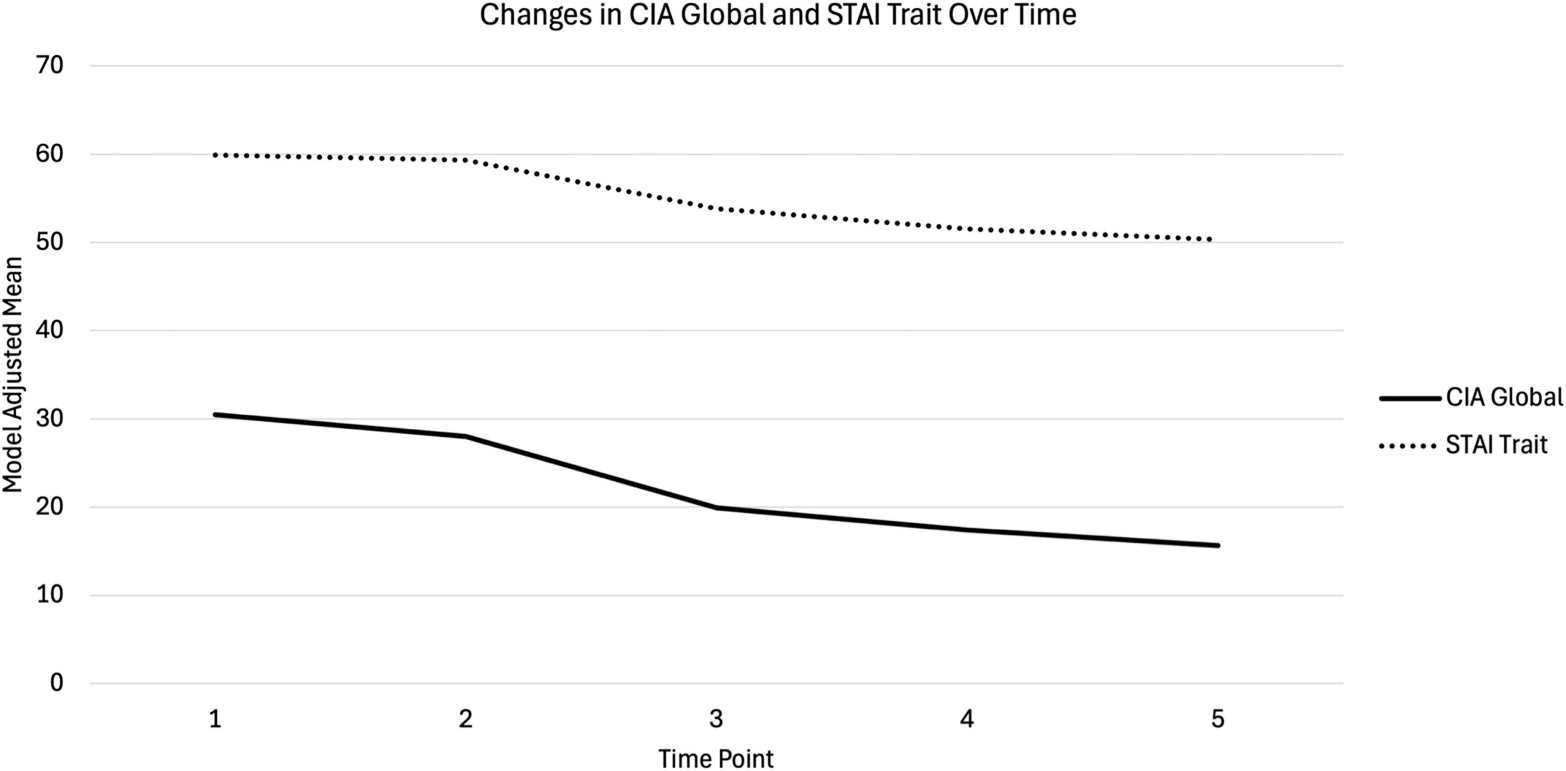
Model-adjusted means for Clinical Impairment Assessment (CIA) Global and State-Trait Anxiety Inventory (STAI)-Trait scores across five time points (Admission, Mid-Treatment, Discharge, 6-Month Follow-Up, and 12-Month Follow-Up). Values represent estimated marginal means from linear mixed-effects models with random intercepts.

Linear mixed-effects modeling revealed significant changes in ED-related impairment over time. Estimated marginal means decreased steadily from Admission (M = 30.54, 95% CI [28.80, 32.28]) to 12MFU (M = 15.64, 95% CI [13.32, 17.96], Figure 2). Pairwise comparisons indicated significant decreases from Admission to all subsequent time points (*p*_admission–mid–treatment_ = 0.047, all other *ps* < 0.0001, *d*_admission–mid–treatment_ = 0.30, *d*_admission–discharge_ = 1.29, *d*_admission–6MFU_ = 1.58, *d*_admission–12MFU_ = 1.80), and from Mid-treatment to Discharge (*p* < 0.0001, *d* = 0.99) 6MFU (*p* < 0.0001, *d* = 1.28), and 12MFU (*p* < 0.0001, *d* = 1.50). No significant differences were observed between Discharge and 6MFU, or between 6MFU and 12MFU, suggesting that ED-related impairment declined most sharply by Discharge and then stabilized at lower levels.

#### Depression symptoms

Linear mixed-effects modeling revealed significant changes in depression over time. Estimated marginal means decreased steadily from Admission (M = 12.94, 95% CI [11.97, 13.91]) to 12MFU (M = 8.52, 95% CI [7.28, 9.67], Figure 1). Pairwise comparisons indicated significant decreases from Admission to Discharge (*p* < 0.0001, *d* = 0.96), 6MFU (*p* < 0.0001, *d* = 1.06), and 12MFU (*p* < 0.0001, *d* = 1.08), and from Mid-treatment to Discharge (*p* < 0.0001, *d* = 0.76), 6MFU (*p* < 0.0001, *d* = 0.86), and 12MFU (*p* < 0.0001, *d* = 0.87). No significant differences were observed between Discharge and 6MFU, or Discharge and 12MFU, or between 6MFU and 12MFU, suggesting that scores declined most sharply by Discharge and then stabilized at lower levels.

#### Anxiety symptoms

Linear mixed-effects modeling revealed significant changes in STAI-Trait scores over time. Estimated marginal means decreased from Admission (M = 59.9, 95% CI [58.3, 61.5]) to 12MFU (M = 50.3, 95% CI [48.3, 52.4], Figure 2). Pairwise comparisons indicated significant decreases from Admission to Discharge (*p* < 0.0001, *d* = 0.90), 6MFU (*p* < 0.0001, *d* = 1.23), and 12MFU (*p* < 0.0001, *d* = 1.40), and from Mid-treatment to Discharge (*p* < 0.0001, *d* = 0.81), 6MFU (*p* < 0.0001, *d* = 1.06), and 12MFU (*p* < 0.0001, *d* = 1.31). No significant differences were observed between Admission and Mid-treatment, between Discharge and 6MFU, or between 6MFU and 12MFU, although a small but significant decrease was observed from Discharge to 12MFU (*p* = 0.005, *d* = 0.50). These results suggested that scores remained relatively stable early in the study, declined sharply by Discharge, and then stabilized at lower levels through 12MFU.

## Discussion

The present study sought to: (1) characterize patients participating in athlete programming during intensive ED treatment, and (2) examine changes in ED, dysfunctional exercise, clinical impairment, depression, and anxiety symptoms throughout treatment. Dysfunctional exercise symptoms decreased significantly early in treatment with medium to large effect sizes observed for pairwise comparisons between Admission and subsequent timepoints. Similarly, ED symptoms demonstrated significant change throughout treatment with effect sizes that ranged from medium to large, with mean values at 12MFU below the clinical cutoff for the EDE-Q (39). Clinical impairment, depression, and anxiety showed similar trajectories with medium to large effect sizes across treatment, patterns indicated sharp declines by mid-treatment and stabilization in later time points. Further, clinical impairment scores at 12MFU were below the clinical cutoff for the CIA indicating clinically meaningful change in respect to impairment secondary to ED features (40). In sum, participation in athlete programming across both adolescent and adult treatment programs was associated with decreases in dysfunctional exercise symptoms without corresponding increases in ED symptoms, ED-related impairment, depression, or anxiety. The results largely supported our hypotheses, indicating that patients participating in athlete programming demonstrated statistically significant improvements across clinical outcomes.

The trajectory of change in dysfunctional exercise mirrors prior work demonstrating that early decreases in compulsive or dysfunctional exercise are predictive of better overall ED outcomes (9). The findings suggest that targeted athlete programming, which incorporates psychoeducation, identity exploration, and structured return-to-sport planning, may be associated with reductions in dysfunctional patterns of exercise that are often deeply entrenched in athletes with EDs. Notably, improvements were not limited to exercise-specific symptoms. Patients demonstrated parallel declines in global ED symptoms, ED-related impairment, depression, and anxiety, with the steepest gains occurring in the first half of treatment (except anxiety, which demonstrated greater decline in the latter half of treatment). These results align with previous research showing that addressing dysfunctional exercise can have broad benefits for ED recovery (8). The maintenance of gains through 6- and 12-month follow-up suggests sustainability of improvements over time, although small non-significant fluctuations at later time points highlight the importance of continued monitoring and post-discharge planning and support.

In addition to demonstrating clinical change, the present study provided preliminary evidence for the feasibility and clinical utility of incorporating athlete-specific programming into intensive ED treatment. In the present study, feasibility was reflected in the integration and delivery of the structured athlete-focused programming within the context of a multi-component partial hospitalization and intensive outpatient treatment program. Symptom scores remained stable or improved across the assessment period within this sample, supporting the safe integration of this programming within a real-world clinical treatment setting. These findings suggest that ED treatment can be adapted to address the unique needs of athletes within routine clinical care. By targeting sport-specific risks such as dysfunctional exercise behaviors, performance pressures, and the psychological complexities of athlete identity, the Athlete group aims to address areas that may not be explicitly emphasized in standard ED programming. This emphasis is clinically meaningful, as athletic identity has been shown to shape both risk for dysfunctional exercise and motivation for recovery (41–43).

The present study was also among the first to systematically evaluate athlete-focused ED treatment in partial hospitalization and intensive outpatient programs, contributing real-world outcomes data across both adolescent and adult programs. These results advance the literature by demonstrating associations between participation in athlete-focused psychoeducational programming and reductions in dysfunctional exercise without corresponding increases in ED psychopathology, impairment, or comorbid symptoms. In doing so, the study strengthens the case for developing and expanding athlete-specific care pathways, which may help establish clearer guidelines for managing exercise and identity-related concerns in ED recovery.

A significant strength of the present study is its longitudinal design with five time points, including a 1-year follow-up from treatment discharge. This duration is essential for assessing long-term patterns of change in individuals receiving interventions targeting dysfunctional exercise symptoms in ED treatment. The use of validated, comprehensive measures, including those assessing ED-related impairment and comorbid mood and anxiety symptoms, further strengthened the findings by offering a broader view of the recovery process and the potential impact of addressing dysfunctional exercise symptoms on ED treatment outcomes.

Despite these strengths, the study was subject to several limitations. First, the absence of a standard-care comparison group of athletes prevented the definitive attribution of observed outcomes to the specialized athlete programming, making it challenging to decouple its unique effects from the benefits of general, evidence-based treatment. Additionally, the present study did not systematically track or control for participants’ exposure to other sport- or exercise-related programming available within the treatment setting. Thus, it is not possible to determine the extent to which observed changes may reflect the broader movement or exercise-related components of treatment that the athlete may have engaged in rather than the athlete programming specifically. This limitation restricts causal interpretation and should be considered when interpreting the findings. Furthermore, this sample was recruited from a higher-level-of-care setting and largely consisted of Non-Hispanic White, female-identifying patients with anorexia nervosa-restricting type as the most common ED diagnosis, which may limit the generalizability of findings to other groups and treatment settings. Additional descriptive information regarding sport type, competitive level, and training context would further strengthen interpretation and generalizability and should be considered in future research. Lastly, a notable limitation of this study was the presence of missing data at later time points, particularly at discharge. Sensitivity analyses indicated that participants with missing data at discharge differed from completers on several baseline variables including admission BMI, baseline levels of ED symptoms, exercise, and ED-related clinical impairment. Although this pattern raised concern for potential non-random missingness, subsequent pattern mixture models suggested that trajectories of change were consistent across groups. These findings provided reassurance that the use of linear mixed-effects models with FIML was appropriate, and that missing data were unlikely to have substantially biased the results. Nevertheless, the possibility of residual bias due to non-random attrition cannot be fully ruled out and should be considered when interpreting findings. Furthermore, although attrition at later time points should be considered when interpreting long-term findings, the overall pattern of results remained stable.

Future research should build upon these initial findings by prioritizing controlled experimental designs, comparing outcomes of those who receive specialized athlete programming versus those receiving standard ED care. Future work should also systematically assess and analytically account for engagement in other exercise- or sport-related interventions, to better understand the unique contribution of the athlete-specific programming. Such work may help clarify the added value of tailoring interventions and investigate the mechanisms through which specialized treatment components (e.g., athlete identity exploration, psychoeducation on RED-S, phased movement reintegration) may be associated with reductions of dysfunctional exercise symptoms. Such research is essential for elucidating and affirming clear, evidence-based guidelines for the treatment of athletes with EDs.

Taken together, these findings highlight the clinical significance of specialized athlete programming within higher levels of ED care. Beyond symptom reduction, such interventions may represent a promising component of ED care for athletes that could foster greater engagement, reduce risk for dropout, and address long-term challenges such as relapse and sport-related injury risk. By demonstrating the feasibility, safety, and potential benefits of athlete-specific programming, this study provides a critical foundation for future evaluations of tailored ED interventions.

## Data Availability

The datasets presented in this article are not readily available because of patient confidentiality and IRB restrictions. De-identified data may be obtained from the corresponding author upon reasonable request and with appropriate institutional approvals. Requests to access the datasets should be directed to Eva-Molly Petitto Dunbar, edunbar@health.ucsd.edu.
