# Supplementary material for: Athlete-focused eating disorder programming in higher levels of care: feasibility and clinical outcomes from a naturalistic setting
Source: Front Nutr. 2026 May 12;13:1734726. doi: 10.3389/fnut.2026.1734726 (PMC13201481; doi:10.3389/fnut.2026.1734726)
Supplement: Supplementary file 1 [file Supplementary_file_1.DOCX]

**Supplementary Material**

***Analysis of Missing Data.*** Initial sensitivity analyses compared participants retained versus missing at each time point on key demographic and clinical variables (Table S1). At time points 2, 4, and 5 missingness varied by some demographic predictors (age, sex, admit BMI, and eating disorder diagnosis; see Table S1), although effect sizes were small. At time point 3 (discharge) participants with missing data differed significantly from those retained, showing moderately higher baseline EDE-Q global, EPSI Excessive Exercise, and CIA scores as well as differences in admit BMI and eating disorder diagnosis (Table S1).

Given these findings, pattern mixture models were conducted to evaluate whether individuals missing at discharge differed in baseline values or longitudinal trajectories for the variables that were deemed to differ (CIA, EDE-Q Global, and EPSI Excessive Exercise) compared to those who completed discharge surveys. For each model, participants were grouped according to whether they were missing data at Time Point 3 (“Missing T3”) or not (“Complete T3”). Each model included fixed effects of Time Point (treated as categorical), Missing T3 status, and their interaction (Time Point × Missing T3), with a random intercept for participant (ID).

**EDE-Q Global**

The main effect indicated that participants missing at Time Point 3 had slightly higher baseline EDE-Q scores compared to completers (*b* = 0.71, *p* = .014).

Interaction terms tested whether trajectories over time differed between the missing and non-missing groups. Results indicated significant differences at later time points: Time Point 4 (*b*=-1.34, *p* = .005), and Time Point 5 (*b=*-1.48*, p* = .004). These estimates were negative, indicating that participants missing at Time 3 showed **larger decreases in EDE-Q global scores** at later assessments compared to those who completed Time 3. Interactions at Time Point 2 were nonsignificant (*b*=.28, *p* = .326).

These findings suggest that while missing-at-Time-3 participants started slightly higher, their trajectories were **not biased upward,** and they tended to improve more than completers by follow-up. This provides reassurance that the use of Linear Mixed-Effects Models with FIML is unlikely to overestimate reductions in EDE-Q scores over time.

**EPSI – Excessive Exercise**

The main effect indicated that participants missing at Time Point 3 had slightly higher baseline EPSI Excessive Exercise scores compared to completers (*b* = 3.50, *p* = .005). However, no significant Time × Missing interactions emerged (*ps*=.087-.696), indicating comparable trajectories between groups across treatment. This suggests that missingness at discharge did not bias the estimated changes in compulsive exercise.

**CIA – Clinical Impairment**

For clinical impairment, there were no differences in baseline CIA scores between groups (*p*=.055) and no significant Time X Missing Interactions (*ps*=.069-.348), indicating comparable trajectories between groups across treatment. This suggests that missingness at discharge did not bias the estimated changes in compulsive exercise.

These results collectively support the appropriateness of using **Linear Mixed-Effects Models with Full Information Maximum Likelihood (FIML)** for handling missing data in the primary analyses.

**Table S1.**

*Characteristics of Individuals with Missing Data Across Timepoints*

| Time Point: 1-Month following Admission | | | |
| --- | --- | --- | --- |
| Variable | Complete  (n = 166) | Missing  (n = 16) | Difference Test/Effect Size |
| Admit Age | 18.72 (6.57) | 22.54 (8.55) | *t*(180) = -2.16, *p* = .032, *d*=-.57 |
| Length of Illness | 4.77 (5.56) | 7.64 (7.05) | *t(*180) = -1.93, *p* = .056 |
| Admit BMI | 19.47 (3.03) | 19.21 (2.80) | *t*(179)= 0.32, *p* =.747 |
| Baseline EPSI Excessive Exercise | 11.04 (6.87) | 12.73 (7.29) | *t*(177)=-0.91, *p* = .364 |
| Baseline EDE-Q Global | 3.54 (1.49) | 3.86 (1.68) | *t*(180)=-0.81, *p* = .422 |
| Baseline Clinical Impairment Assessment | 30.31 (11.10) | 32.06 (12.94) | *t*(177)=-0.59, *p* = .553 |
| Baseline PHQ-9 | 12.85 (6.58) | 13.20 (7.49) | *t*(176)=-0.19, *p* = .849 |
| Baseline STAI-Trait | 59.91 (10.30) | 59.50 (12.26) | *t*(178)=0.15, *p* = .882 |
| ED Diagnosis |  |  | *c^2^*(4) = 3.99, *p* = .408 |
| AN-R | 92 | 6 |  |
| AN-BP | 26 | 2 |  |
| BN | 12 | 3 |  |
| ARFID | 9 | 1 |  |
| OSFED | 27 | 4 |  |
| Length of Stay | 113.88 (37.55) | 101.15 (57.42) | *t*(12.88)= 1.12, *p* =.447 |
| Sex |  |  | *c^2^*(1) = 3.29, *p* = .070 |
| Female | 153 | 12 |  |
| Male | 17 | 4 |  |
| Race |  |  | *c^2^*(2) = 5.12, *p* = .077 |
| White | 145 | 12 |  |
| Asian | 8 | 3 |  |
| Other | 15 | 1 |  |
| Ethnicity |  |  | *c^2^*(1) = 0.35, *p* = .852 |
| Hispanic | 24 | 2 |  |
| Non-Hispanic | 145 | 14 |  |
| Time Point: Discharge | | | |
| Variable | Complete  (n = 151) | Missing  (n = 31) | Difference Test/Effect Size |
| Admit Age | 19.20 (7.12) | 18.35 (5.20) | *t*(180) = 0.63, *p* = .532 |
| Length of Illness | 4.06 (6.14) | 4.83 (3.21) | *t*(180) =0.21, *p* = .835 |
| Admit BMI | 19.21 (3.02) | 20.57 (2.68) | *t*(179)= -2.31, *p* =.022, *d*=-.46 |
| Baseline EPSI Excessive Exercise | 10.58 (6.89) | 14.13 (6.31) | *t*(177)=-2.61, *p* = .010, *d*=-.52 |
| Baseline EDE-Q Global | 3.46 (1.52) | 4.14 (1.30) | *t*(180)=-2.32, *p* = .021, *d*=-.46 |
| Baseline Clinical Impairment Assessment | 29.67 (11.21) | 34.26 (10.86) | *t*(177)=-2.08, *p* = .039, *d*=-.41 |
| Baseline PHQ-9 | 12.64 (6.53) | 14.03 (7.16) | *t*(176)=-1.06, *p* = .292 |
| Baseline STAI-Trait | 59.53 (10.04) | 61.52 (12.27) | *t*(178)=-0.96, *p* = .337 |
| ED Diagnosis |  |  | *c^2^*(4) = 23.76, *p* <.001 *V*=.36 |
| AN-R | 89 | 9 |  |
| AN-BP | 25 | 3 |  |
| BN | 11 | 4 |  |
| ARFID | 9 | 1 |  |
| OSFED | 17 | 14 |  |
| Length of Stay | 113.63 (37.55) | 106.35 (53.77) | *t*(17.80)= 0.54, *p* = .594 |
| Sex |  |  | *c^2^*(1) = 2.42, *p* = .120 |
| Female | 140 | 25 |  |
| Male | 15 | 6 |  |
| Race |  |  | *c^2^*(2) = 1.16, *p* = .560 |
| White | 133 | 24 |  |
| Asian | 8 | 3 |  |
| Other | 13 | 3 |  |
| Ethnicity |  |  | *c^2^*(1) = 4.26, *p* = .039 *V*=.152 |
| Hispanic | 18 | 8 |  |
| Non-Hispanic | 136 | 23 |  |
| Time Point: 6-Month Follow-Up (Post-Discharge) | | | |
| Variable | Complete  (n = 99) | Missing  (n = 83) | Difference Test/Effect Size |
| Admit Age | 18.27 (4.82) | 19.98 (8.58) | *t*(123.82) =-1.61, *p* = .109 |
| Length of Illness | 4.36 (4.45) | 5.81 (6.93) | *t*(134.96) = -1.64, *p* = .104 |
| Admit BMI | 19.16 (2.96) | 19.80 (3.03) | *t*(179)= -1.44, *p* =.152 |
| Baseline EPSI Excessive Exercise | 10.54 (7.02) | 11.94 (6.73) | *t*(177)=-1.36, *p* = .176 |
| Baseline EDE-Q Global | 3.74 (1.45) | 3.37 (1.54) | *t*(180)=1.68, *p* = .093 |
| Baseline Clinical Impairment Assessment | 30.72 (11.62) | 30.15 (10.84) | *t*(177)=0.34, *p* = .736 |
| Baseline PHQ-9 | 13.54 (6.42) | 12.08 (6.86) | *t*(176)=1.46, *p* = .146 |
| Baseline STAI-Trait | 60.68 (10.31) | 58.89 (10.60) | *t*(178)=1.14, *p* = .254 |
| ED Diagnosis |  |  | *c^2^*(4) = 6.85, *p* = .144 |
| AN-R | 61 | 37 |  |
| AN-BP | 15 | 13 |  |
| BN | 6 | 9 |  |
| ARFID | 5 | 5 |  |
| OSFED | 12 | 19 |  |
| Length of Stay | 109.46 (41.16) | 117.81 (36.33) | *t*(166)= -1.36, *p* = .177 |
| Sex |  |  | *c^2^*(1) = 6.88, *p* = .009, *V*=.192 |
| Female | 97 | 68 |  |
| Male | 6 | 15 |  |
| Race |  |  | *c^2^*(2) = 1.56, *p* = .460 |
| White | 90 | 67 |  |
| Asian | 5 | 6 |  |
| Other | 7 | 9 |  |
| Ethnicity |  |  | *c^2^*(1) = 0.42, *p* = .516 |
| Hispanic | 16 | 10 |  |
| Non-Hispanic | 87 | 72 |  |
| Time Point: 12-Month Follow-Up (Post-Discharge) | | | |
| Variable | Complete  (n = 78) | Missing  (n = 104) | Difference Test/Effect Size |
| Admit Age | 18.22 (4.60) | 19.67 (8.08) | *t*(169.05) =- 1.53, *p* = .127 |
| Length of Illness | 4.18 (4.40) | 5.65 (6.53) | *t*(180) = -1.72, *p* = .088 |
| Admit BMI | 18.93 (2.76) | 19.84 (3.13) | *t*(174.75)=-2.08, *p* =.039, *d*=-.31 |
| Baseline EPSI Excessive Exercise | 10.21 (7.02) | 11.89 (6.77) | *t*(177)=-1.62, *p* = .107 |
| Baseline EDE-Q Global | 3.72 (1.48) | 3.46 (1.52) | *t*(180)=1.14, *p* = .255 |
| Baseline Clinical Impairment Assessment | 30.28 (11.42) | 30.61 (11.18) | *t*(177) = -0.19, *p* = .848 |
| Baseline PHQ-9 | 12.73 (6.05) | 13.01 (7.08) | *t*(176) = -0.28, *p* = .781 |
| Baseline STAI-Trait | 60.21 (10.28) | 59.62 (10.62) | *t*(178) = 0.37, *p* = .709 |
| ED Diagnosis |  |  | *c^2^*(4) = 9.79, *p* = .044, *V*=.23 |
| AN-R | 51 | 47 |  |
| AN-BP | 10 | 18 |  |
| BN | 3 | 12 |  |
| ARFID | 5 | 5 |  |
| OSFED | 9 | 22 |  |
| Length of Stay | 108.73 (37.27) | 116.50 (40.93) | *t*(166)= -1.28, *p*=.203 |
| Sex |  |  | *c^2^*(1) = 5.55, *p* = .019, *V*=.17 |
| Female | 76 | 89 |  |
| Male | 4 | 17 |  |
| Race |  |  | *c^2^*(2) = 0.02, *p* = .990 |
| White | 68 | 89 |  |
| Asian | 5 | 6 |  |
| Other | 7 | 9 |  |
| Ethnicity |  |  | *c^2^*(1) = 0.15, *p* = .701 |
| Hispanic | 12 | 14 |  |
| Non-Hispanic | 67 | 92 |  |

*Note.* AN-R = anorexia nervosa, restricting subtype; AN-BP = anorexia nervosa, binge/purge subtype; BMI = Body Mass Index; BN = bulimia nervosa; EDE-Q = Eating Disorder Examination—Questionnaire; OSFED = other specified feeding or eating disorder; PI = Pacific Islander

**Table S2.**

*Fit statistics for Pattern Mixture Models*

| Model | *S-BX^2^* | *df* | *b* | *p* | RMSEA | CFI |
| --- | --- | --- | --- | --- | --- | --- |
| Unconditional Model of Compulsive Exercise | |  |  |  |  |  |
|  | 11.76 | 5 | 4.66* | .038 | .06 | .99 |
| Conditional Model with Exercise Identity | |  |  |  |  |  |
|  | 14.05 | 7 | 4.58* | .050 | .06 | .99 |
| Conditional Model with Exercise Identity X Peer Norms | | | |  |  |  |
|  | 17.95 | 11 | 3.11* | .083 | .04 | .99 |

Note: Abbreviations AIC= Akaike Information Criteria.
